# Transcriptional Profiles Elucidate Differential Host Responses to Infection with *Cryptococcus neoformans* and *Cryptococcus gattii*

**DOI:** 10.3390/jof8050430

**Published:** 2022-04-22

**Authors:** Zachary E. Holcomb, Julie M. Steinbrink, Aimee K. Zaas, Marisol Betancourt, Jennifer L. Tenor, Dena L. Toffaletti, J. Andrew Alspaugh, John R. Perfect, Micah T. McClain

**Affiliations:** 1 Harvard Combined Dermatology Residency Program, Department of Dermatology, Massachusetts General Hospital, Boston, MA 02114, USA; zholcomb@partners.org; 2 Division of Infectious Diseases and International Health, Department of Medicine, Duke University Medical Center, Durham, NC 27710, USA; aimee.zaas@duke.edu (A.K.Z.); mbetaqui@gmail.com (M.B.); jennifer.tenor@duke.edu (J.L.T.); dena.toffaletti@duke.edu (D.L.T.); andrew.alspaugh@duke.edu (J.A.A.); john.perfect@duke.edu (J.R.P.); micah.mcclain@duke.edu (M.T.M.); 3 Department of Molecular Genetics and Microbiology, Duke University School of Medicine, Durham, NC 27710, USA; 4 Infectious Diseases Section, Medical Service, Durham Veteran’s Affairs Medical Center, Durham, NC 27705, USA

**Keywords:** *Cryptococcus*, transcriptomics, diagnostics

## Abstract

Many aspects of the host response to invasive cryptococcal infections remain poorly understood. In order to explore the pathobiology of infection with common clinical strains, we infected BALB/cJ mice with *Cryptococcus neoformans*, *Cryptococcus gattii*, or sham control, and assayed host transcriptomic responses in peripheral blood. Infection with *C. neoformans* resulted in markedly greater fungal burden in the CNS than *C. gattii*, as well as slightly higher fungal burden in the lungs. A total of 389 genes were significantly differentially expressed in response to *C. neoformans* infection, which mainly clustered into pathways driving immune function, including complement activation and TH2-skewed immune responses. *C. neoformans* infection demonstrated dramatic up-regulation of complement-driven genes and greater up-regulation of alternatively activated macrophage activity than seen with *C gattii*. A 27-gene classifier was built, capable of distinguishing cryptococcal infection from animals with bacterial infection due to *Staphylococcus aureus* with 94% sensitivity and 89% specificity. Top genes from the murine classifiers were also differentially expressed in human PBMCs following infection, suggesting cross-species relevance of these findings. The host response, as manifested in transcriptional profiles, informs our understanding of the pathophysiology of cryptococcal infection and demonstrates promise for contributing to development of novel diagnostic approaches.

## 1. Introduction

Cryptococcal species trigger invasive fungal infections that often result in life-threatening disease. The vast majority of illness from cryptococcal species is due to *Cryptococcus neoformans* (*C. neoformans*), but the closely related species *Cryptococcus gattii* (*C. gattii*) is an emerging pathogen in some geographic areas. The most devastating manifestation of cryptococcal infection is cryptococcal meningitis, with over 200,000 cases occurring worldwide each year [1,2]. The clinical manifestations of cryptococcosis often overlap with many other infectious pathogens, which can make clinical diagnosis of cryptococcal infection challenging. 

*C. neoformans* and *C. gattii* trigger disease states that share many similarities. Both species are capable of causing pulmonary cryptococcosis as well as cryptococcal meningitis and meningoencephalitis. However, *C. neoformans* is generally considered to be an opportunistic pathogen that infects primarily immunocompromised hosts, while *C. gattii* is more commonly isolated from immunocompetent hosts [3]. Clinically, *C. gattii* is more likely to cause pulmonary infection and less likely to infect the central nervous system than *C. neoformans* [4]. In terms of the host side of the infection equation, both intact innate and adaptive immune systems are necessary for optimal response to cryptococcal infection [5]. Studies have shown roles for complement components and anti-capsular antibodies in the phagocytosis of cryptococcal organisms and outlined the critical nature of T_H_1 polarization in successful immune responses [6,7,8,9,10,11,12,13]. Classically activated M1 macrophages that result from T_H_1 immune profiles are important for clearance of *Cryptococcus*, while alternatively activated M2 macrophages resulting from T_H_2 cytokine stimulation are associated with increased pathogenicity [14,15]. Natural killer cells are also important in the clearance of *Cryptococcus*, both through direct cryptococcal clearance and through promotion of a T_H_1-mediated immune response [16,17,18]. 

The gold standard for diagnosis is culture of cryptococcal organisms, but low sensitivity and prolonged time to results can delay important treatment decisions. Other diagnostic tests, including India ink staining and cryptococcal antigen detection, may sacrifice accuracy for low turnaround time [19,20,21]. Despite these available tests, diagnosis of this devastating disease in a timely manner often remains a challenge, and improved diagnostics would be a welcome addition to our armamentarium. One promising diagnostic niche that is rapidly evolving involves the identification of pathogen-specific, host-based gene expression changes in circulating white blood cells responding to infection [22,23,24,25,26,27,28]. There are data suggesting that fungal organisms trigger unique pathogen class-specific transcriptomic responses during *Candida albicans* infection [29], but, as yet, the utility of this approach for cryptococcosis has not been explored.

In order to further elucidate the nature of the host response to these organisms, we examined peripheral blood transcriptional changes in response to infection with *C. gattii* and *C. neoformans* in a mouse model. This has permitted exploration of the biology of the host response to cryptococcal challenge, the differentiation of the responses to two important cryptococcal species, and the development of a gene-expression based classifier of acute cryptococcal infection.

## 2. Materials and Methods

### 2.1. Inoculum Preparation

*C. gattii* R265 and *C. neoformans* H99 strains were used in this study. Each strain was incubated in yeast extract peptone dextrose (YPD) medium for 48 h in a shaking incubator (220 rpm) with adequate aeration at 30 °C. An amount of 10 milliliters of the culture was pelleted (3000 rpm) in 50-milliliter conical tubes for 5 min and washed twice with 10 milliliters of PBS at a pH of 7.4. The cells were resuspended in 1 milliliter of phosphate-buffered saline (PBS) and counted with a hemacytometer. The cell concentration was adjusted to 6 × 10^5^ cells/milliliter with PBS and mice were inoculated with a total of 1.5 × 10^4^ cells in a volume of 25 microliters.

### 2.2. C. gattii and C. neoformans Infection

This study was carried out in strict accordance with the recommendations in the Guide for the Care and Use of Laboratory Animals of the National Institutes of Health. All protocols for murine work (A178-14-07) were approved by the Duke Institutional Animal Care and Use Committee and the Duke Office of Animal Welfare Assurance (OAWA). A total of 45 mice (BALB/cJ, female, weighing 17 to 21 g, age 8 weeks, The Jackson Laboratory) were separated into three experimental groups. One group of 15 mice received intranasal inoculation of 1.0 × 10^4^–1.5 × 10^4^ *C. neoformans* cells suspended in 25 microliters of PBS. A second group of 15 mice received intranasal inoculation of 1.0 × 10^4^–1.5 × 10^4^ *C. gattii* cells suspended in 25 microliters of PBS. The control group of 15 mice received intranasal inoculation of 25 µL of the vehicle (PBS). The mice were weighed at the time of initial inoculation and daily during the second week of the experiment to assess clinical status. After 14 days post-infection, the mice were sacrificed for collection of whole blood via cardiac puncture. Blood (500 microliters) was placed in RNAlater tubes provided in the Mouse RiboPure RNA Isolation kit per the manufacturer’s (Ambion, Austin, TX, USA) instructions. WBC counts and manual cell differentials (using 100 cells) were performed on the blood from cardiac puncture. The lungs, brain, and spleen tissues of infected mice were collected into sterile PBS, weighed, homogenized, serially diluted, and plated on YPD agar for colony count.

### 2.3. RNA Preparation

Whole blood RNA isolation and β-globin reduction were carried out on 30 samples (10 from each experimental group) using the manufacturer’s protocol (Mouse RiboPure and GLOBINclear, Ambion). The amount and purity of RNA yield was analyzed using the NanoDrop spectrophotometer (Thermo Fischer Scientific, Waltham, MA, USA) and the integrity was analyzed using the Agilent Bioanalyzer. RNA from 28 of the 30 samples that met quality control checks (260/280 ratio > 1.8, 260/230 ratio > 1.0, and RNA integrity number > 7) were used for microarray analysis. RNA was amplified and biotin-labeled using MessageAmp Premier RNA Amplification kit (Ambion) according to standard protocols at the Duke University Microarray Core facility. The Duke University Microarray Core performed amplification and hybridization onto Affymetrix murine 430A2.0 microarrays. Probe intensities were detected using Axon GenePix 4000B Scanner (Molecular Devices, San Jose, CA, USA). Image files were generated using Affymetrix GeneChip Command Console software.

### 2.4. Statistical Analysis

Weight change and WBC counts were compared between the three experimental groups using Kruskal–Wallis one-way ANOVA. Pairwise comparisons of fungal tissue burden and white blood cell differentials between experimental groups were performed utilizing the Wilcoxon signed-rank test. For gene expression quantification, Affymetrix microarray data were initially processed, underwent quality control checks, and were normalized with the robust multi-array average method using the *affy* [30] Bioconductor [31] package from the R statistical programming environment [32]. Differential expression was carried out using a moderated t-statistic from the *limma* package [33]. The false discovery rate was used to control for multiple hypothesis testing.

### 2.5. Pathway Analysis

Differentially regulated pathways and gene ontology terms were identified using Ingenuity Pathway Analysis (IPA) [34] and the Database for Annotation, Visualization, and Integrated Discovery (DAVID) [35,36]. The significance of association between the differentially expressed genes in the data set and the canonical pathway was measured by Fisher’s exact test and by the ratio of genes from the data set that mapped to the pathway divided by the total number of genes in the pathway. Significance was reported as a *p*-value.

In order to determine up- or down-regulation of an individual pathway, Fisher’s exact test was used to compare the proportion of genes that were up-regulated and the proportion of genes that were down-regulated compared to baseline expression levels in healthy control mice. Similarly, to compare preferential activation of one pathway over another, Fisher’s exact test was used to compare the proportion of genes that were appropriately up- or down-regulated in each pathway compared to baseline expression levels in healthy control mice.

### 2.6. Classifier Development

A Lasso logistic regression model was employed on the normalized expression microarray data using the *glmnet* [37] package from the R statistical programming environment [32]. All probesets that matched that either didn’t match to a gene or matched to a gene on a sex chromosome were eliminated prior to the regression. We tested our algorithm for generating predictive models of infection with *Cryptococcus* species using 100 repetitions of 10-fold cross-validation, resulting in a range of possible predictions for each sample depending on which of the remaining samples were used to build the predictive model (probability). 

### 2.7. Validation of Findings in Human PBMCs

Additional validation was performed with an in vitro PBMC microarray dataset consisting of viral (influenza), bacterial (*Escherichia coli* and *Streptococcus pneumoniae*), and fungal (*Candida albicans*, and *Cryptococcus neoformans* and *gattii*) infections of healthy human PBMCs. Whole blood was drawn from six healthy individuals (3 males, 3 females: ages 25–35) through the Duke Healthy Donor Research Protocol, and PBMCs were isolated via a standard Ficoll gradient procedure. Cells were then re-suspended in RPMI 5 and plated in duplicate at a concentration of 6 × 10^6^ cells per well into 24-well plates. Relevant pathogens or controls were then added at different concentrations (influenza viruses at a final concentration of 10^3^TCID_50_, LPS 1 ug/mL, Poly I:C 5 ug/mL, *Streptococcus pneumoniae* and *Escherichia coli* at 10^5^ per well, *Candida albicans*, *Cryptococcus neoformans*, and *Cryptococcus gattii* at 10^6^ per well). Bacteria and fungi were heat-killed prior to exposure to human cells to prevent microbe-induced killing of the PBMCs. Cells were then incubated at 37 °C with 5% CO_2_ for 24 h, at which time cells were harvested and underwent centrifuge purification from culture media. Cells were washed and placed in Qiagen RLT lysis buffer per the manufacturer’s instructions. RNA was then extracted and hybridized, and microarray data collection was performed at Expression Analysis (Durham, NC, USA) using the GeneChip^®^ Human Genome U133A 2.0 Array (Affymetrix, Santa Clara, CA, USA).

## 3. Results

A total of 45 BALB/cJ mice were divided into three experimental groups: *C. gattii* (R265) infection, *C. neoformans* (H99) infection, and healthy controls. In prior experience with this model, the mice typically succumb to lethal effects of infection between three and four weeks. Based on this timeline, an intermediate timepoint was selected for the current study (mice were sacrificed at 14 days post-inoculation), when mice would have developed disseminated infection but not yet be presenting with severely morbid disease. After intranasal inoculation, there was no significant difference in weight among the groups (*p* = 0.07), overall level of clinical disease was moderate, and none of the mice met preset criteria for early euthanasia during the course of the experiment. 

### 3.1. Mice Infected with C. neoformans Develop More Severe Fungal Burden

**Figure 1:**
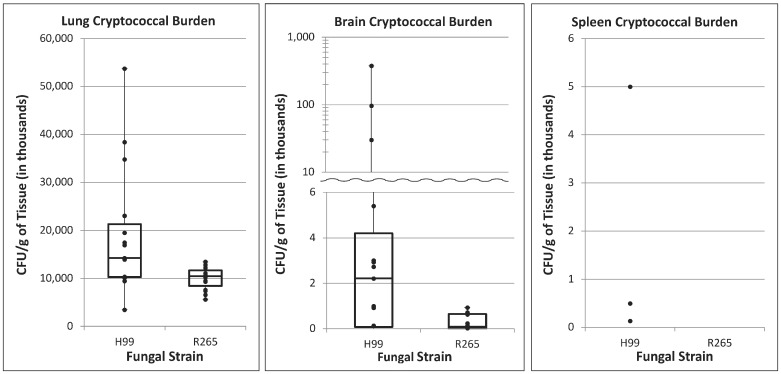
**Fungal tissue burden in lungs, brain, and spleen.** Fungal burden in lung, brain, and spleen tissues was measured for the two cohorts of infected mice at the time of sacrifice 14 days after inoculation.

Fungal burden in lung, brain, and spleen tissues was measured for the two cohorts of infected mice at the time of sacrifice 14 days after inoculation (Figure 1). *C. neoformans* infection resulted in increased burden of disease in the lungs compared to *C. gattii* (median 1.43 × 10^7^ CFU/g in H99 vs. 1.05 × 10^7^ CFU/g in R265; *p* = 0.01) and markedly increased tissue burden in the brain as well (median of 2.22 × 10^3^ CFU/g in H99 vs. 8.98 × 10^2^ CFU/g in R265; *p* = 0.02). For splenic tissue, only three samples had fungal burden above the lower limit of detection (<10 CFU/g), all of which contained *C. neoformans* organisms. Although tissue collected from mice infected with both *C. gattii* and *C. neoformans* showed strong evidence of infection and fungal dissemination, the tissue burden was consistently heavier for mice infected with *C. neoformans*. 

Peripheral blood drawn by cardiac puncture at the time of sacrifice (14 days) was also examined for evidence of fungal organisms. Interestingly, 10 of the 15 blood cultures in the *C. gattii* cohort grew fungal organisms, while only 5 of the 15 cultures in the *C. neoformans* cohort grew fungi. Therefore, despite the finding that fungal tissue burden was greater in the lungs, brain, and spleen in *C. neoformans* infection, *C. gattii* infection was associated with a greater degree of fungemia at the time of sampling. Interestingly, Ngamskulrungroj et al., similarly found that *C. neoformans* produced more central nervous system invasive disease than *C. gattii*. However, in contrast, *C. gattii* was not recovered from blood and did not grow well in serum in that model. The reason for our differences is not clear but may reside in variation present between the two studies in timing of collection, mouse background, or inoculum [38].

### 3.2. Peripheral White Blood Cell Differentials of Mice Infected with Both C. gattii and C. neoformans Differ from Control Mice

**Figure 2:**
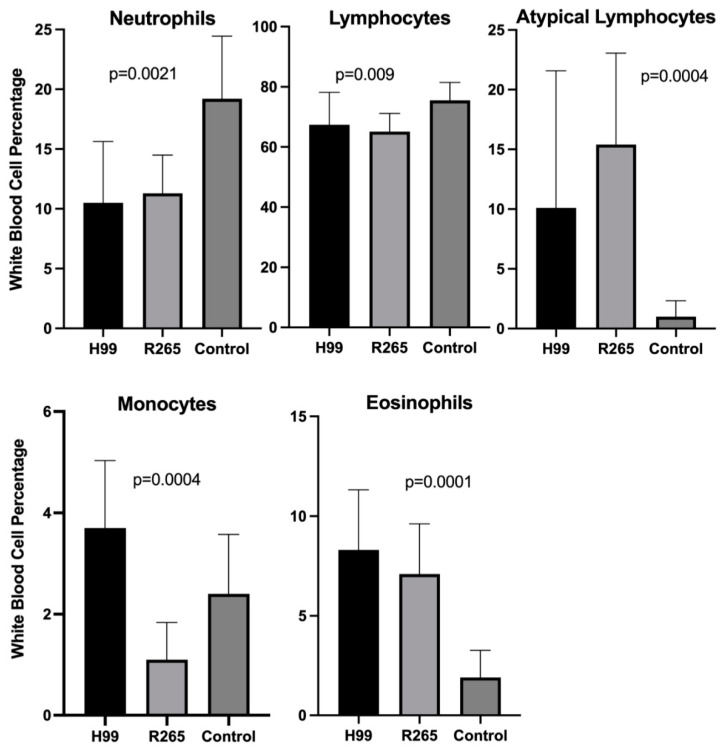
**Variation in circulating white blood cells.** Mean levels of each cell type for *C. neoformans* (H99), *C gattii* (R265), and controls in peripheral blood samples taken at day 14 post-inoculation. Error bars indicate standard deviation. *p* values denote significant differences between groups.

Despite minimal clinical symptoms of infection due to the relatively brief 14-day course of the experiment, a number of significant perturbations in laboratory variables, such as peripheral white blood cell (WBC) counts, were observed. Mean total peripheral WBC counts were not significantly different between the groups (*p* = 0.6). WBC subset analysis, however, demonstrated marked perturbations compared to healthy controls (Figure 2 and Table 1). Mice infected with *Cryptococcus* had significantly higher levels of circulating atypical lymphocytes (10.1% in *C. neoformans* infection, 15.4% in *C. gattii* infection, and 1.0% in controls) and eosinophils (8.3% in *C. neoformans* infection, 7.1% in *C. gattii* infection, and 1.9% in controls) in peripheral blood, while percentages of neutrophils and typical lymphocytes were significantly lower in infected animals. The cohort infected with *C. neoformans* showed increased circulating monocytes compared to healthy controls, while mice infected with *C. gattii* had fewer monocytes circulating in peripheral blood than healthy controls.

### 3.3. Mice Infected with Cryptococcus Exhibit a Powerful Transcriptomic Response to Infection with Many Broadly Conserved Components Regardless of Fungal Species

**Figure 3:**
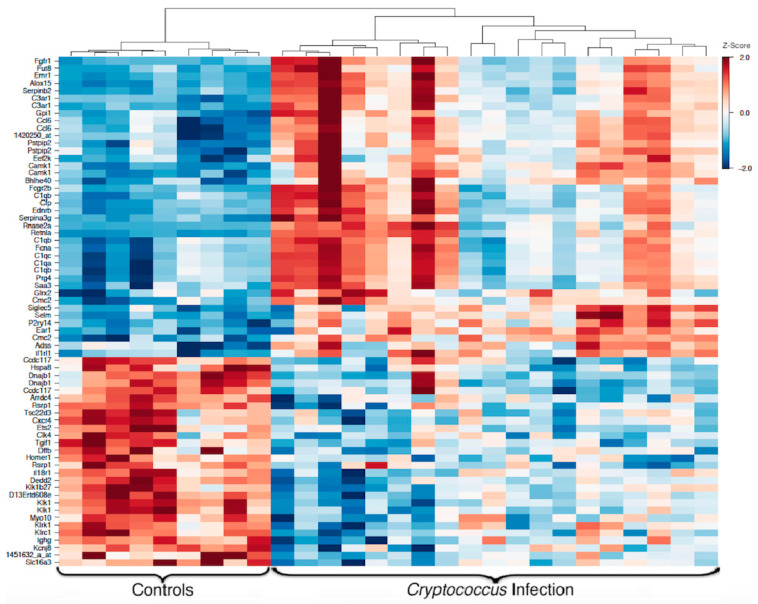
**Heat map of genes differentially expressed between infected and healthy mice.** Solid red indicates a Z-score of 2.0 or greater, while solid blue indicates a Z-score of −2.0 or lower. Gene names are listed on the left, while clustering is indicated at the top of the figure.

Transcriptomic responses of peripheral blood immune cells exhibit marked changes in response to *Cryptococcus* infection. When grouped together and compared to healthy controls, mice infected with *C. gattii* or *C. neoformans* had 3426 significant differentially expressed genes (*p* < 0.05), and 87 of these genes had a 2-fold change or greater from baseline expression levels in healthy mice. After correcting for multiple testing, 68 genes were found to be significantly differentially expressed (*p* < 0.05) between infected mice and controls, with 25 of these genes exhibiting at least a 2-fold change from baseline. Of the 68 significant differentially expressed genes, 40 were up-regulated in the infected mice, while 28 were down-regulated in response to infection (Figure 3). While there was some variability in the expression of each of these 68 genes within the infected group, the overall similarity in the response to infection permits strong clustering by infection status.

The most differentially expressed genes in mice infected with *Cryptococcus*, in terms of fold change as compared to healthy mice, are shown in Table 2. The majority of these differentially expressed genes function in known pathways relating to immunologic responses. In particular, of the top 10 genes with the largest change in response to cryptococcal infection, three are components of the classical complement activation pathway: *C1qb*, *C1qa*, and *C1qc*. Additionally, *Rnase2a*, *Retnla*, and *Chil3* are components of T_H_2-mediated immune pathways, and *Serpinb2* has been shown to suppress the T_H_1 arm of the immune response [39,40,41,42,43,44,45,46]. Therefore, the most strongly up-regulated genes provide evidence of complement-mediated immunity and an adaptive, T_H_2-skewed immune response to infection with *Cryptococcus*.

Pathway analysis of the most significantly up-regulated genes in response to cryptococcal infection also primarily revealed clustering of these genes into pathways related to immune function (Table 3), including “immune response,” “acute inflammatory response”, and “complement activation”. In addition, several of the most significant pathways relating to the up-regulated genes represented B cell immune function, including “humoral immune response”, “immunoglobulin-mediated immune response”, and “B cell-mediated immunity”. However, the significant genes that mapped to these B cell pathways were mostly complement components, including *C1qa*, *C1qb*, *C1qc*, and *Cfp*. Therefore, the significance of B cell pathway enrichment likely represents antibody-mediated complement activation, rather than other aspects of B cell activity.

**Figure 4:**
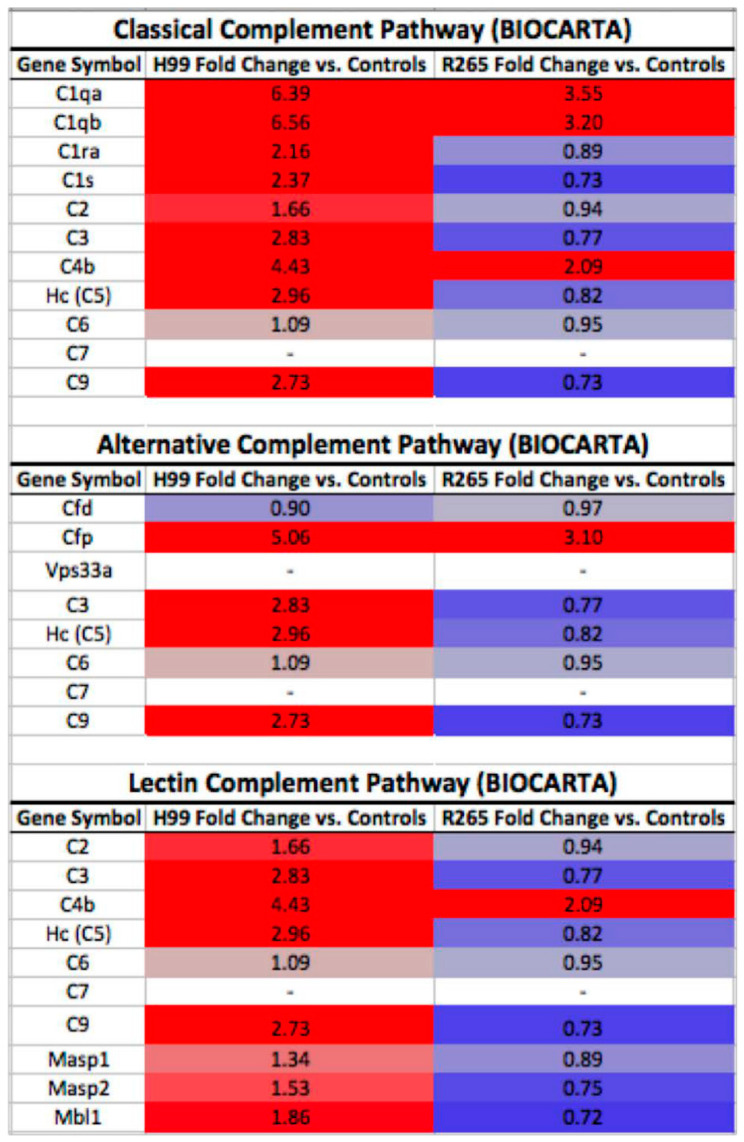
**Alteration in expression of genes involved in the classical, alternative, and lectin complement activation pathways.** Relative fold change for each gene due to infection with *C. gattii* R265 and *C. neoformans* H99 in comparison to healthy control mice. Genes listed are from BIOCARTA-defined pathways, with solid red representing 2-fold up-regulation and solid blue representing 2-fold down-regulation. Genes with no fold change listed did not have probes on the microarray panel.

As previous studies have demonstrated, our data show that complement plays a major role in the immune response to infection by both *C. gattii* and *C. neoformans* [47,48]. Examination of quantitative changes in expression of the genes of the classical, alternative, and lectin complement activation pathways shows that genes from all three pathways are highly up-regulated in mice infected with *C. neoformans* (Figure 4). For mice with *C. gattii* infection, certain components of the complement pathway are heavily up-regulated, primarily within the classical complement activation pathway, but these are notably muted compared to the up-regulation seen in *C. neoformans*.

Although many of the most significantly up-regulated host genes and functional pathways in response to infection with *Cryptococcus* are related to complement activation, we also see evidence of skewing of the adaptive immune response. Transcriptomic evidence of a T_H_2-mediated immune response is shown above in Table 2, where 3 of the 10 most up-regulated genes in infected mice have known roles in T_H_2 immunity (*Rnase2a*, *Retnla*, and *Chil3*). In addition, *Serpinb2*, another of the most heavily up-regulated genes, has been shown to actively suppress a T_H_1 immune response. Therefore, genes that were most differentially expressed from baseline provide strong evidence of a T_H_2 mediated immune response. 

In addition to finding differentially expressed genes that are directly involved in T_H_2-mediated immunity, up-regulation of certain macrophage-related genes also pointed toward the presence of a T_H_2-skewed response. Alternatively activated M2 macrophages promote T_H_2 immune responses, and products of a T_H_2 immune response down-regulate M1 macrophage activity [49]. Certain genes that are up- and down-regulated in classically activated (M1) and alternatively activated (M2) macrophages have been identified [50]. 

### 3.4. Differences in the Responses to C. gattii and C. neoformans Are Reflected in the Host Transcriptome

The most powerful gene expression changes in response to infection with *Cryptococcus*, including up-regulation of complement activity and evidence of a T_H_2 immune response, are shared between mice infected with *C. gattii* and mice infected with *C. neoformans*. However, subtle differences in the transcriptomic response were also identified between infection with the two species. The most noticeable difference between the murine responses to *C. gattii* and *C. neoformans* infection was the magnitude of gene expression change observed. Compared to uninfected controls, mice infected with *C. gattii* exhibited 39 genes that were differentially expressed in response to infection, including 10 with at least a 2-fold change from baseline. In comparison, *C. neoformans* infection induced significant differential expression of 389 genes, with 67 of those showing at least a 2-fold change compared to control mice. Furthermore, the most differentially expressed gene in mice infected with *C. gattii* was *Serpinb2* with a +5.5-fold change from baseline, while the most differentially expressed gene in response to *C. neoformans* infection, *Rnase2a*, had a +41-fold change from baseline. Overall, these data support a more robust immune response, as measured at the transcriptomic level, in response to *C. neoformans* infection. These notable differences in the transcriptome following *C. neoformans* infection suggest that this more robust immune response is necessary to combat a species more known for its dissemination and neuroinvasive tendencies.

Differences in the magnitude of the transcriptomic response between species are further evident in complement activation pathways. As shown in Figure 4, there was a larger degree of induction of complement-related genes in response to *C. neoformans* infection across all three complement activation pathways. Despite up-regulation of certain genes within each pathway in response to *C. gattii* infection, comparison of the proportion of up-regulated genes between the two infected cohorts revealed significantly greater transcriptional activity of the classical, alternative, and lectin pathways for mice infected with *C. neoformans* (*p*-values of 0.001, 0.004, and 0.002, respectively).

In addition to more transcriptionally active complement pathways, mice infected with *C. neoformans* also showed greater up-regulation of genes involved in M2 macrophage activation and M1 macrophage suppression [50]. For *C. gattii* infection, the degree of M2 activation on the gene expression level was not significantly greater than the degree of M1 activation (*p* = 0.1781). On the other hand, there were significant changes in *C. neoformans* (*p* = 0.0002), which favored M2 activation, demonstrating more powerful M2 polarization and therefore a more skewed T_H_2 immune response.

Aside from differences in complement activation and the degree of T_H_2-mediated immunity through M2 macrophage activation, there were other, less prominent fungal species-specific differences noted as well. Out of the 28 genes found to be significantly down-regulated in response to *C. neoformans* infection, eight probes corresponding to six unique genes (*Klrc1*, *Pak1*, *Ighg*, *Klrk1*, *Fasl*, and *Klra7*) were associated with natural killer cell-mediated cytotoxicity (*p* = 0.006). Previous studies have indicated the importance of natural killer cells in the successful eradication of *C. neoformans* [51,52,53,54], suggesting that suppression of natural killer cell function may contribute to fungal dissemination in the host as seen in our model.

### 3.5. A Transcriptomic Classifier Accurately Identifies Cryptococcal Infection

**Figure 5:**
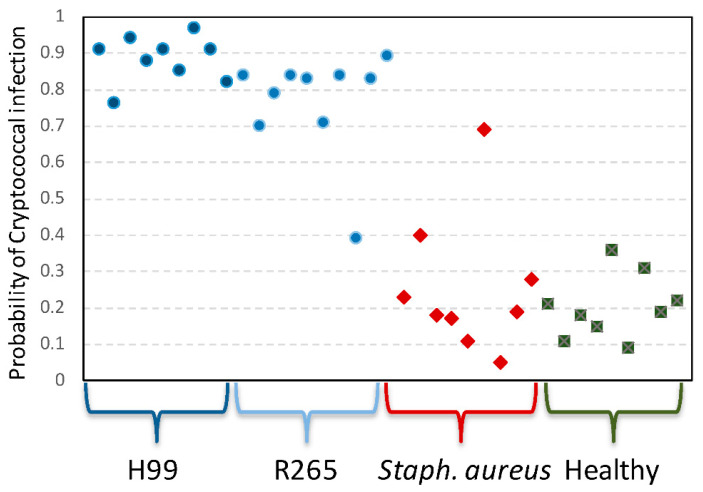
**Performance of a 27-gene transcriptomic signature of cryptococcal infection.** Depicted are calculated probabilities of cryptococcal infection for individual mice infected with *C. neoformans* (“H99”), *C gattii* (“R265”), *Staphylococcus aureus*, or uninfected control animals (“Healthy”).

In addition to elucidating the pathobiology of infectious processes, patterns of differentially expressed genes in the host can also be utilized to identify the presence of infection, and, potentially, to differentiate types of infectious agents. Based on differentially expressed genes in our experimental model, a binomial classifier that differentiated infected and healthy mice was produced. The classifier consisted of 28 probes representing 27 unique genes. Genes contained in the classifier included: *Cmc2*, *Ear1*, *Il1rl1*, *Klk1*, *Slc16a3*, *Dedd2*, *Dnajb1*, *Ccl6*, *Camk1*, *Zfp704*, *Dffb*, *Prkg2*, *P2ry14*, *Prrx1*, *Klra19*, *Hspa8*, *Rab3c*, *Cwf19l1*, *Arhgap29*, *D6Ertd160e*, *Neu1*, *Rsrp1*, *Cxcr4*, *Retnla*, *Ccdc117*, *Arap2*, and *Mela* (Supplemental Table S1). When applied to all samples from the experiment, the 27-gene binomial classifier had 100% accuracy in differentiating infected mice from healthy controls. We next examined the performance of this classifier in an existing microarray dataset from our prior work [29], and the cryptococcal classifier was also capable of differentiating cryptococcal infection from murine bacterial infection (with *Staphylococcus aureus*) with 94% sensitivity and 89% specificity (Figure 5).

### 3.6. Translation to Human Applications

To better understand how transcriptional responses identified in these data translate across species, the top genes differentially expressed in the murine response to *Cryptococcus* (as compared to controls) in this study were compared to the top differentially expressed genes seen when human PBMCs were subjected to yeast exposure in vitro (*Candida albicans*, *Cryptococcus neoformans*, and *Cryptococcus gattii*). When examining overlapping responses in the top differentially expressed genes, 73 genes were noted to be significantly expressed (*p* < 0.05) in both systems (Supplemental Table S2). Additionally, when specifically examining human orthologs of the 27 genes of the murine cryptococcal classifier, 6 of the 14 genes (43%) in which data were available were also significantly expressed in human cells upon exposure to these pathogens. These data suggest that the observed gene expression changes are not isolated to the murine species. This degree of species overlap is similar to our previously published *Candida* data [29,55], where an overlap of less than 50% of the differentially expressed genes between mice and humans still allowed for highly accurate performance of the classifier in human hosts.

## 4. Discussion

Using an intranasal inoculation model in mice, we have, for the first time, examined specifically the host response to *C. neoformans* and *C. gattii* infection as manifest through patterns of gene expression in circulating white blood cells. Conserved changes in host gene expression were evident in response to *C. gattii* and *C. neoformans* infection, although the magnitude of these conserved host responses was much greater in animals infected with *C. neoformans*. Furthermore, transcriptional analysis allows for description of unique differences between the biological responses elicited by these two species, and discriminatory models based on this differential expression can be used to develop novel classifiers that offer the potential to diagnose this challenging disease. 

Alterations at the transcriptomic level reflect the pathophysiology of the way the host responds to these invading organisms. Early on in infection, elements of the complement cascade have been demonstrated to play a significant role in the host response to both *C. gattii* and *C. neoformans* [6,48,56,57]. This has even been demonstrated at the transcriptional level in other tissues and cell culture models, although not directly in circulating leukocytes [58,59,60]. This response was observed in our model as well, with genes involved in the classical, alternative, and lectin activation pathways up-regulated in response to both *C. neoformans* and *C. gattii* infection. However, all three complement pathways were much more significantly up-regulated in response to *C. neoformans* infection than to *C. gattii* infection, again reflecting the stronger response to *C. neoformans* in our experimental model. Previous work has demonstrated that mice deficient in *C1q* show overwhelming pulmonary fungal burden with *C. gattii* [48]. In our model, *C1qa* and *C1qb* were two of the top 10 most differentially up-regulated genes in response to *C. gattii* infection, providing additional evidence of their important role in the immune response to this fungal species.

Many of the most significantly up-regulated genes in response to *Cryptococcus* infection were either products of a T_H_2 immune response (*Rnase2a*, *Retnla*, *Chil3*) or negative regulators of a T_H_1 immune response (*Serpinb2*). Furthermore, there was evidence of alternatively activated M2 macrophage responses on the transcriptomic level, which are associated with a T_H_2-type immune response. When comparing the two infected cohorts, the host response to *C. neoformans* was significantly more skewed toward T_H_2-mediated immunity than the host response to *C. gattii*. Since a T_H_2-skewed immune response has been linked to greater fungal burden and extra-pulmonary dissemination [7,8,9,10], the stronger T_H_2 transcriptomic polarization seen in *C. neoformans* infection may partially explain the greater fungal burden found in the lungs, brain, and splenic tissue with this species. This may also explain some of the propensity of *C. neoformans* to infect HIV-positive patients, whose immune profiles tend to change from T_H_1 to being T_H_2-biased over time [61]. Additionally, mechanisms postulated to promote survival and extra-pulmonary dissemination of *C. neoformans* include eicosanoid production via arachidonic acid acquired from the host [62] and suppression of natural killer cell activity [51,52,53,54]. As described above, we discovered transcriptomic changes in the *C. neoformans* cohort that supported activation of both of these processes, linking their occurrence to fungal dissemination in the host.

*C. gattii* has a greater propensity for causing clinical disease in immunocompetent hosts. One suggested mechanism for this is the ability of *C. gattii* to suppress adaptive immune system recognition through suppression of antigen presentation on antigen-presenting cells and subsequent T lymphocyte proliferation [63]. We observed transcriptomic evidence of this mechanism of immune evasion occurring in the *C. gattii* cohort, as reflected by the down-regulation of genes in antigen processing and presentation pathways. Down-regulation of similar pathways were not observed in response to *C. neoformans* infection, raising the possibility that this absence of active immune evasion partially contributes to the more robust immune response to *C. neoformans* infection on the gene expression level. 

In addition to providing insight into the pathophysiology and mechanistic responses to cryptococcal infection, analysis of differentially expressed genes in the infected state also shows promise for the development of pathogen class-specific diagnostics. By building a classifier composed of a small number of unique differentially expressed genes, infected and healthy mice could be differentiated with 100% accuracy. This same classifier was capable of accurately discriminating *Cryptococcus*-infected animals from animals with acute bacterial infection due to *Staphylococcus aureus*. Furthermore, we observed many of these same genes and pathways were activated (or suppressed) when human PBMCs were infected with the same species of *Cryptococcus*, which also overlaps with signals described during cryptococcal infection of human monocytes in vitro [64]. These preliminary findings are limited by the need to use heat-killed microbial strains for co-culture with the human PBMCs to prevent overt host cell toxicity by proliferating fungi and bacteria. However, despite these limitations, these studies suggest that findings from our murine studies may also be applicable to human disease [64]. As we have shown in prior work, sparse gene-based signatures such as these also lend themselves well to migration to more clinic-ready RT-PCR platforms, some of which are already commonly present in clinical microbiology laboratories [65].

Although peripheral blood gene expression analysis provides a wealth of information into the host response to infectious challenge, this approach has clear limitations. Peripheral blood immune effector cells are only one component of the broader immune response, and gene expression changes in sampled cells do not reflect the entirety of the host response. In terms of biological significance, genomic studies are a critical component of our armamentarium, but they are ultimately hypothesis-generating in scope, and future directed biological experiments are necessary to confirm preliminary conclusions about functional immune mechanisms. The experimental model utilized is limited by the collection of peripheral blood at only one time point, thereby precluding analysis of the transcriptomic response in later, more clinically severe infection. We chose the time point of 14 days after initial inoculation to allow for establishment of infection and dissemination within the host, while simultaneously avoiding clinical deterioration and gene expression changes associated with overwhelming illness and death, which tend to occur between three and four weeks in this infection model. While in our prior work early transcriptomic signatures offer even better diagnostic performance at the time of maximal clinical disease [25,66], we did not have the opportunity to definitively assess this in the current work. Future studies of how these types of responses are manifest in patients with clinical disease and under varying levels of immunosuppression will be critical to proving relevance of these data, and any cryptococcal disease classifier will require validation in other populations (clinical syndromic mimics, immunosuppressed hosts, etc.) in order to determine true clinical relevance.

We have demonstrated robust blood-based transcriptomic responses in the host during cryptococcal infection, including those which are conserved across species, as well as genomic pathways that differ in both character and magnitude between *C. gattii* and *C. neoformans*. This study highlights the ability of host peripheral blood gene expression analysis to elucidate the underlying biology of infection. Additionally, given the widening availability of clinic-ready RT-PCR based platforms capable of measuring gene expression in clinical samples, these findings lay the groundwork for development of diagnostic assays based on changes in host-derived biomarkers of cryptococcal infection. 

## Figures and Tables

**Table 1:** Differences in peripheral white blood cell counts.

Leukocyte Subtype	H99	R265	Controls	*p*-Values (** = Statistically Significant)
Neutrophils	10.5%	11.3%	19.2%	H99 vs. R265 (*p* = 0.8786) **H99 vs. Controls (*p* = 0.0055)** ** R265 vs. Controls (*p* = 0.0014) **
Lymphocytes	67.4%	65.1%	75.5%	H99 vs. R265 (*p* = 0.2552) H99 vs. Controls (*p* = 0.0636) **R265 vs. Controls (*p* = 0.0025)** **
Atypical Lymphocytes	10.1%	15.4%	1.0%	H99 vs. R265 (*p* = 0.1033) **H99 vs. Controls (*p* = 0.0119)** ** R265 vs. Controls (*p* = 0.0001) **
Monocytes	3.7%	1.1%	2.4%	**H99 v. R265 (*p* = 0.0296)** ** H99 v. Control (*p* = 0.0116) ** R265 v. Control (*p* = 0.0004) **
Eosinophils	8.3%	7.1%	1.9%	H99 vs. R265 (*p* = 0.3803) **H99 vs. Controls (*p* = 0.0003)** ** R265 vs. Controls (*p* = 0.0004) **

**Table 2:** Top 10 up-regulated genes in mice infected with *Cryptococcus* compared to controls.

Gene	Fold-Change (Infected vs. Control)	Function
*Rnase2a*	+14.15	Produced in response to T_H_2 cytokine stimulation and serves as a macrophage chemoattractant [39,46].
*Retnla*	+6.85	Produced by macrophages in response to IL-4 and T_H_2-mediated inflammation [41]. Inhibits T_H_2-mediated immunity and inflammation [42].
*Serpinb2*	+5.77	Expressed by myeloid antigen-presenting cells and plays a role in suppression of the T_H_1 immune response [45].
*C1qb*	+5.37	Component of the classical pathway of complement activation.
*Chil3*	+5.35	Produced by macrophages in response to IL-4 and T_H_2-mediated inflammation [41].
*Alox15*	+5.26	Involved in arachidonic acid metabolism [40]
*C1qa*	+4.76	Component of the classical pathway of complement activation.
*Prg4*	+4.65	Produces a glycoprotein for articular joint protection and PTH-responsive hematopoiesis and megakaryopoiesis [43].
*C1qc*	+4.48	Component of the classical pathway of complement activation.
*Ear1*	+4.33	Produces a host defense protein secreted by eosinophils, macrophages, and neutrophils [44].

**Table 3:** The 10 most significant biological process GO terms that correspond to the 40 genes up-regulated in cryptococcal infection.

GO Term	Bonferroni-Corrected *p*-Value
Immune response (GO:0006955)	0.00002
Acute inflammatory response (GO:0002526)	0.00389
Complement activation (GO:0006956)	0.01135
Activation of plasma proteins involved in acute inflammatory response (GO:0002541)	0.01135
Innate immune response (GO:0045087)	0.01166
Immune effector process (GO:0002252)	0.02201
Defense response (GO:0006952)	0.03399
Humoral immune response (GO:0006959)	0.03808
Immunoglobulin mediated immune response (GO:0016064)	0.05967
B cell mediated immunity (GO:0019724)	0.06529

## Data Availability

Primary microarray datasets analyzed in this work will be uploaded to the National Center of Biotechnology Information’s Gene Expression Omnibus upon acceptance for publication.

## Supplementary Materials

The following supporting information can be downloaded at: https://www.mdpi.com/article/10.3390/jof8050430/s1, Table S1: Genes of the *Cryptococcus* Classifier; Table S2: Genes significantly expressed (*p* < 0.05) in both PBMC (*Candida* and *Cryptococcus*) and Murine (*Cryptococcus*) Datasets.
