# Supplementary material for: Transcriptional Profiles Elucidate Differential Host Responses to Infection with Cryptococcus neoformans and Cryptococcus gattii
Source: J Fungi (Basel). 2022 Apr 22;8(5):430. doi: 10.3390/jof8050430 (PMC9143552; doi:10.3390/jof8050430)
Supplement: Supplementary file 1 [file jof-08-00430-s001.zip › jof-1692371-supplementary.pdf]

Supplemental Table S1. Genes of the *Cryptococcus* Classifier

**Genes**
Cmc2
Ear1
Il1rl1
Klk1
Slc16a3
Dedd2
Dnajb1
Ccl6
Camk1
Zfp704
Dffb
Prkg2
P2ry14
Prrx1
Klra19
Hspa8
Rab3c
Cwf19l1
Arhgap29
D6Ertd160e
Neu1
Rsrp1
Cxcr4
Retnla
Ccdc117
Arap2
Mela

Supplemental Table S2. Genes significantly expressed ($p<0.05$) in both PBMC (*Candida* and *Cryptococcus*) and Murine (*Cryptococcus*) Datasets

Gene Symbol	Adjusted P Value PBMC Dataset	Adjusted P Value Murine Dataset
**Cmc2**	0.00120	0.00444
**Il18r1**	0.00157	0.01886
**Bhlhe40**	0.00017	0.02290
**Myo10**	0.0000003	0.02433
**Cmc2**	0.00120	0.02648
**Mphosph9**	0.00202	0.03017
**Dnajb1**	0.02913	0.03017
**Tnfrsf1b**	0.03787	0.03062
**Dag1**	0.00170	0.03342
**Glrx2**	0.00245	0.03342
**Tnfrsf25**	0.04066	0.03342
**Rsrp1**	0.00700	0.03494
**Cxcr3**	0.01345	0.03832
**Ets2**	0.01437	0.03968
**Clcf1**	0.01101	0.03981
**Arih2**	0.00001	0.03987
**Fkbp11**	0.00251	0.03987
**Creld2**	0.02583	0.03987
**Tsc22d3**	0.01757	0.04088
**Plxnc1**	0.00017	0.04170
**Pld1**	0.00202	0.04170
**Creb3l2**	0.02475	0.04170
**Il2rb**	0.00103	0.04240
**Smad7**	0.01079	0.04268
**Ccl24**	0.00045	0.04321
**Slc20a1**	0.01626	0.04355
**Dag1**	0.00170	0.04548
**Il10ra**	0.02082	0.04548
**Dpagt1**	0.00225	0.04565
**Nagk**	0.00552	0.04592
**Fut8**	0.00666	0.04592
**Rps6kb2**	0.02114	0.04791
**Dnajb1**	0.02913	0.04850
**Clk1**	0.00116	0.04853
**Ddb2**	0.00128	0.04853

**Il18**	0.00004	0.04908
**Spry2**	0.00001	0.04908
**Nid1**	0.00002	0.04908
**Tnrc6b**	0.00004	0.04908
**Emc2**	0.00009	0.04908
**Dnajb6**	0.00113	0.04908
**Ctla4**	0.00176	0.04908
**Pld1**	0.00202	0.04908
**Tgfbr1**	0.00238	0.04908
**Cryl1**	0.00341	0.04908
**Adcy3**	0.00369	0.04908
**Jarid2**	0.00716	0.04908
**Unc45a**	0.00763	0.04908
**Spn**	0.01271	0.04908
**Atp2a2**	0.01273	0.04908
**Hdgf**	0.01459	0.04908
**Snapc3**	0.01471	0.04908
**St3gal6**	0.01722	0.04908
**Hspa5**	0.01791	0.04908
**Arhgef2**	0.03035	0.04908
**Clk4**	0.00006	0.04911
**Impad1**	0.02438	0.04937
**Myc**	0.000005	0.04989
**Txndc9**	0.00009	0.04989
**Morf4l2**	0.00046	0.04989
**Cept1**	0.00078	0.04989
**Ddb2**	0.00128	0.04989
**Smyd2**	0.00555	0.04989
**Atp5o**	0.00587	0.04989
**Prkacb**	0.00746	0.04989
**Ufsp2**	0.00774	0.04989
**Clcf1**	0.01101	0.04989
**Dhx30**	0.01175	0.04989
**Stk17b**	0.01218	0.04989
**Sel1l**	0.01690	0.04989
**Plcb1**	0.02065	0.04989
**Dennd2d**	0.03638	0.04989
**Pde8a**	0.03645	0.04989
